# The Inhibitory Effect of Lysophosphatidylcholine on Proangiogenesis of Human CD34^+^ Cells Derived Endothelial Progenitor Cells

**DOI:** 10.3389/fmolb.2021.682367

**Published:** 2021-06-10

**Authors:** Haijun Zhao, Yanhui He

**Affiliations:** ^1^ Department of Pain, The First Hospital of Jilin University, Changchun, China; ^2^ Department of Ophthalmology, The Second Hospital of Jilin University, Changchun, China

**Keywords:** LPC, EPC, CD31, angiogenesis, cell biology

## Abstract

Increasing evidence reveals that lysophosphatidylcholine (LPC) is closely related to endothelial dysfunction. The present study aimed to investigate the mechanism of LPC in inhibiting the proangiogenesis and vascular inflammation of human endothelial progenitor cells (EPCs) derived from CD34^+^ cells. The early EPCs were derived from CD34^+^ hematopoietic stem cells whose purity was identified using flow cytometry analysis. The surface markers (CD34, KDR, CD31; VE-cadherin, vWF, eNOS) of EPCs were examined by flow cytometry analysis and immunofluorescence. RT-qPCR was used to detect the mRNA expression of inflammatory cytokines (CCL2, IL-8, CCL4) and genes associated with angiogenesis (VEGF, ANG-1, ANG-2) in early EPCs after treatment of LPC (10 μg/ml) or phosphatidylcholine (PC, 10 μg/ml, control). The angiogenesis of human umbilical vein endothelial cells (HUVECs) incubated with the supernatants of early EPCs was detected by a tube formation assay. The mRNA and protein levels of key factors on the PKC pathway (phosphorylated PKC, TGF-β1) were measured by RT-qPCR and western blot. The localization of PKC-β1 in EPCs was determined by immunofluorescence staining. We found that LPC suppressed the expression of CCL2, CCL4, ANG-1, ANG-2, promoted IL-8 expression and had no significant effects on VEGF expression in EPCs. EPCs promoted the angiogenesis of HUVECs, which was significantly inhibited by LPC treatment. Moreover, LPC was demonstrated to promote the activation of the PKC signaling pathway in EPCs. In conclusion, LPC inhibits proangiogenesis of human endothelial progenitor cells derived from CD34^+^ hematopoietic stem cells.

## Introduction

The endothelial progenitor cells (EPCs) derived from hematopoietic stem cells of bone marrow is the precursor of endothelial cells. EPCs contribute to endothelialization and vascularization via the secretion of vasoactive and angiogenic factors including VEGF, HGF, Ang-1, SDF-1α, TGF-β, and IL-8 to repair the damaged tissues (Rosenzweig, 2003; Li and Li, 2016). EPCs have been reported to protect differentiated endothelial cells from apoptosis, while the repair mechanism of EPCs was weakened in the aging process (Wang et al., 2019). The dysfunction and loss of EPCs are dominant characteristics of many vascular pathologies. Increasing attention has been drawn in the application of EPCs as regenerative medicine in vascular pathologies such as cardiovascular diseases, atherosclerosis, and thrombosis (Liu et al., 2009; Li and Li, 2016; Kou et al., 2020). However, some limitations of EPC transplantation such as emboli formation, immunogenicity, and malignant transformation hinder the application of EPCs in stem cell therapy (Herberts et al., 2011).

Lysophosphatidylcholine (LPC) is the primary component of oxidized low-density lipoprotein (ox-LDL) which is a major contributor to many cardiovascular diseases (Itabe et al., 1994; Tsimikas et al., 2003). Previous studies have revealed that LPC promotes inflammation (Sharma et al., 2020), endothelial dysfunction (Kugiyama et al., 1990), injury and apoptosis of vascular smooth muscle cells (Hsieh et al., 2000). The expression of LPC is altered in many pathologies. For example, LPC expression is significantly elevated in the plasma of psoriatic patients (Zeng et al., 2017). LPC is a potent atherogenic molecule with an 8-fold upregulated expression levels in the intima and inner media of atherosclerotic aorta of squirrel monkeys and controls the incorporation of fatty acid into aortic phospholipid in atherosclerosis (Portman and Alexander, 1969). Rheumatoid arthritis is related to increased LPC expression that is positively correlated with cardiovascular risks (Giraud et al., 2019). Moreover, LPC and sphingolipids showed significant expression alteration in patients with acute aortic dissection and are suggested to be used as potential biomarkers for its diagnosis (Zhou et al., 2019).

The influence of oxidized low-density lipoprotein (oxLDL) on EPCs has been revealed by a previous study that oxLDL inhibits the cell growth, migration and adhesiveness of EPCs [13]. The oxLDL or LPC facilitates apoptosis and inhibits survival of EPCs by suppressing the PI3K/Akt signaling pathway and downregulating the expression of endothelial nitric oxide synthase (eNOS) (Ma et al., 2006; Tie et al., 2010; Hong et al., 2015), which may lead to various diseases (Liu et al., 2020). However, the underlying mechanisms between LPC and EPCs remained uninvestigated.

The aim of this study was to explore the function of LPC on EPCs derived from CD34^+^ hematopoietic stem cells *in vitro*. We hypothesized that LPC exerts suppressive effect on EPC angiogenesis and the underlying mechanism was further investigated, which may provide therapeutic basis for the application of EPC transplantation in the treatment of various vascular diseases.

## Materials and Methods

### Cell Culture and Identification

The study was approved by the ethics committee of the Second Hospital of Jilin University (approval number: 2021-061; Jilin, China). All participants gave informed consents before the study. The human hematopoietic stem cells were isolated from peripheral blood (80 ml) of healthy adult donors (*n* = 12). The peripheral blood was diluted with PBS in a ratio of 1:3. Subsequently, 20 ml of the peripheral blood was added into the Lymphocyte Separation Medium (Ficoll-Pague T Plus, 10 ml) and centrifuged at 400 g for 30 min. A pasteur pipette was used to transfer the middle layer of mononuclear cells into a centrifuge tube. Peripheral blood mononuclear cells were washed with 40 ml of PBS and mechanically dissociated into single cell suspension. Next, Magic™ Fc Receptor Blocker (Abace Biotechnology) and 100 μL of magnetic beads of CD34^+^ was added into the cell suspension and incubated for 30 min at 4°C. Finally, CD34^+^ cells were collected using MS Columns and MiniMACS™ Separator. The early EPCs were differentiated from CD34^+^ human hematopoietic stem cells. The CD34^+^ cells at the density of 1 × 10^6^ cells/cm^2^ were seeded on fibronectin-coated dish and cultured at 37°C in 5% CO_2_. The FACSCalibur™ flow cytometer (Becton, Dickinson and Company, Singapore) was used to detect the labeled EPCs in the peripheral blood and a CellQuest™ software (BD Biosciences) was used for quantification. Common endothelial cell lines used for the scientific research include human umbilical vein endothelial cells (HUVECs), bovine aortic endothelial cells and microvascular endothelial cells. HUVECs have the advantage of human origin, compared with the bovine aortic endothelial cells, and are relatively easier to be cultured, compared with the microvascular endothelial cells. HUVECs were purchased from American Type Culture Collection and incubated with Dulbecco’s modified Eagle’s medium (DMEM; Thermo Fisher, Shanghai, China) containing 10% FBS at 37°C in 5% CO_2_. EPCs received three different treatments: 1) PBS, 2) 10 μg/ml of LPC, 3) 10 μg/ml of phosphatidylcholine (PC, the negative control).

### Flow Cytometry Analysis

The suspension of EPCs (200 μL) were added with 3 ml of PBS at 4°C in a closed falcon tube. Then 10 µL of polyclonal antibodies against CD34, KDR and CD31 labeled with FITC were added into the tubes. After coincubation at 4°C for 1 h in the dark, PBS was used to wash the EPCs followed by centrifugation and removal of the supernatant. After resuspending in PBS with 0.5% paraformaldehyde, CD34^+^ hematopoietic stem cells and CD34^+^, KDR^+^, CD31^+^ EPCs were quantified in triplicate relative to the number of CD45^+^FS_high_SS_high_ granulocytes in the sample with a FACSCalibur™ flow cytometer by a CellQuest™ software (BD Biosciences).

### Reverse Transcription Quantitative Polymerase Chain Reaction

Total RNAs in EPCs were isolated by TRIzol reagent (Invitrogen). The concentration of the extracted RNAs was assessed using the NanoDrop ND-1000 spectrophotometer (Thermo Fisher Scientific), and the absorbance was read at 260 and 280 nm. Reverse transcription of total RNA (1 µg) into complementary DNA was conducted with a RevertAid First Strand cDNA Synthesis Kit (Thermo Fisher) according to the manufacturer’s instructions. A QuantStudio five Real-Time PCR System (Thermo Fisher) was used to perform the qPCR assays under the thermocycling conditions as follows: 40 cycles at 95°C for 15 s, at 60°C for 1 min. The 2^−∆∆Ct^ method (Livak and Schmittgen, 2001) was used to quantify the relative mRNA levels of CCL2, IL-8, CCL4, VEGF, ANG-1, ANG-2, PKC-β1, TGF-β1 normalized to GAPDH. The primer sequences are provided in Table 1.

**TABLE 1:** The primer sequences used for RT-qPCR.

Targets	Forward (5′-3′)	Reverse (5′-3′)
CCL2	CCA​GAT​GCA​ATC​AAT​GCC​C	TGG​TCT​TGA​AGA​TCA​CAG​CT
IL-8	TGGCAGCCTTCCTGATTT	AACCCTCTGCACCCAGTT
CCL4	CAG​CTG​TGG​TAT​TCC​AAA​CC	CAT​ACA​CGT​ACT​CCT​GGA​CC
VEGF	CTTGCTGCTCTACCTCCA	AAATGCTTTCTCCGCTCT
ANG-1	AACCGAGCCTATTCACAG	AAGCATCAAACCACCATC
ANG-2	AAT​TAT​TCA​GCG​ACG​TGA​GG	GAA​GGG​TTA​CCA​AAT​CCC​AC
PKC-β1	CGT​CCT​CAT​TGT​CCT​CGT​A	GCA​CAG​GCA​CAT​TGA​AGT​A
TGF-β1	CTG​TGG​CTA​CTG​GTG​CTG​AC	CAT​AGA​TTT​CGT​TGT​GGG​TTT​C
GAPDH	TCA​TTT​CCT​GGT​ATG​ACA​ACG​A	GTC​TTA​CTC​CTT​GGA​GGC​C

### Western Blot

Proteins in EPCs were extracted using RIPA lysis buffer (Invitrogen) followed by centrifugation at 12,000 g for 15 min at 4°C. The ultraviolet spectroscopy was used to determine the concentrations of proteins. After being isolated with 10% sodium dodecyl sulfate polyacrylamide gel electrophoresis, 50 µg of proteins were transferred to a nitrocellulose membrane which was blocked with 5% non-fat milk at room temperature for 1 h. Then the membranes were incubated with primary antibodies including anti-CCL2 (#ab214819, 1/1,000, Abcam), anti-CCL4 (#ab45690, 1/1,000, Abcam), anti-IL-8 (#ab235584, 1/1,000, Abcam), anti-VEGF (#ab1316, 1/2000, Abcam), anti-ANG-1 (#ab8451, 1/500, Abcam), anti-ANG-2 (#ab155106, 1/500, Abcam), anti-TGF-β1 (#ab215715, 1/1,000, Abcam), anti-p-PKC (#ab109539, 1/1,000, Abcam), anti-GAPDH (#ab9485, 1/2,500, Abcam) at 4°C overnight. GAPDH served as the loading control. Next, membranes were incubated with horseradish peroxidase conjugated secondary antibodies for 1 h at room temperature. The enhanced chemiluminescence solution (Thermo Fisher Scientific) was used to visualize the targeted protein bands. The ImageJ software was used to detect densitometry analysis of the band intensity.

### Immunofluorescence Staining

Immunofluorescence staining was used to detect the expression of EPC surface markers or the location of PKC-β1 in EPCs. EPCs (1 × 10^5^ cells per well) were fixed on 8-well chamber slides with 4% paraformaldehyde for 15 min, and then blocked with 0.1% bovine serum albumin for 30 min at room temperature followed by incubation with the primary antibodies including anti-VE-cadherin (#ab225443, 1/50, Abcam), anti-vWF (#195029, 1/50, Abcam), anti-Enos (#ab76198, 1/200, Abcam), anti-PKC-β1 (#ab223452, 1/250, Abcam) at 4°C overnight. On the second day, cells were incubated with fluorescence (Alexa Fluor 488 or Alexa Fluor 647) labeled secondary antibodies for 1 h at 37°C. Subsequently, EPCs were stained with diamidino-phenyl-indole for 10 min at 37°C in the dark. A fluorescence microscope, Olympus FV 1000 (Olympus, Seoul, Korea), was used to capture the images.

### Tube Formation Assay

Each well of the 96-well plates was precoated with 50 μL of Matrigel (Corning, Bedford, MA, United States). EPCs (1 × 10^5^ cells per well), control HUVECs (2 × 10^4^ cells per well), HUVECs (2 × 10^4^ per well) under different treatments: 1) 100 μL of culture supernatant of EPC cultures; 2) 100 μL of culture supernatant of EPC cultures and 10 μg/ml of PC; 3) 100 μL of culture supernatant of EPC cultures and 10 μg/ml of LPC were plated into 96-well plates at 37°C with 1% O_2_ and 5% CO_2_ for 10 h. The tube counts and capillary lengths in each group were observed in five randomly chosen visual fields at ×100 magnification using ImageJ software.

### Statistical Analysis

Data were shown as the mean ± standard deviation. SPSS23.0 was used for statistical analyses. Normality was tested using a Shapiro-Wilk normality test. The comparison between two groups was evaluated by Student’s t-test, and difference among multiple groups was evaluated by one-way analysis of variance followed by Tukey’s post hoc test. All experiments were performed triplicates independently. *p* < 0.05 was regarded as statistically significant.

## Results

### Identification of CD34^+^ Hematopoietic Stem Cells

**FIGURE 1:**
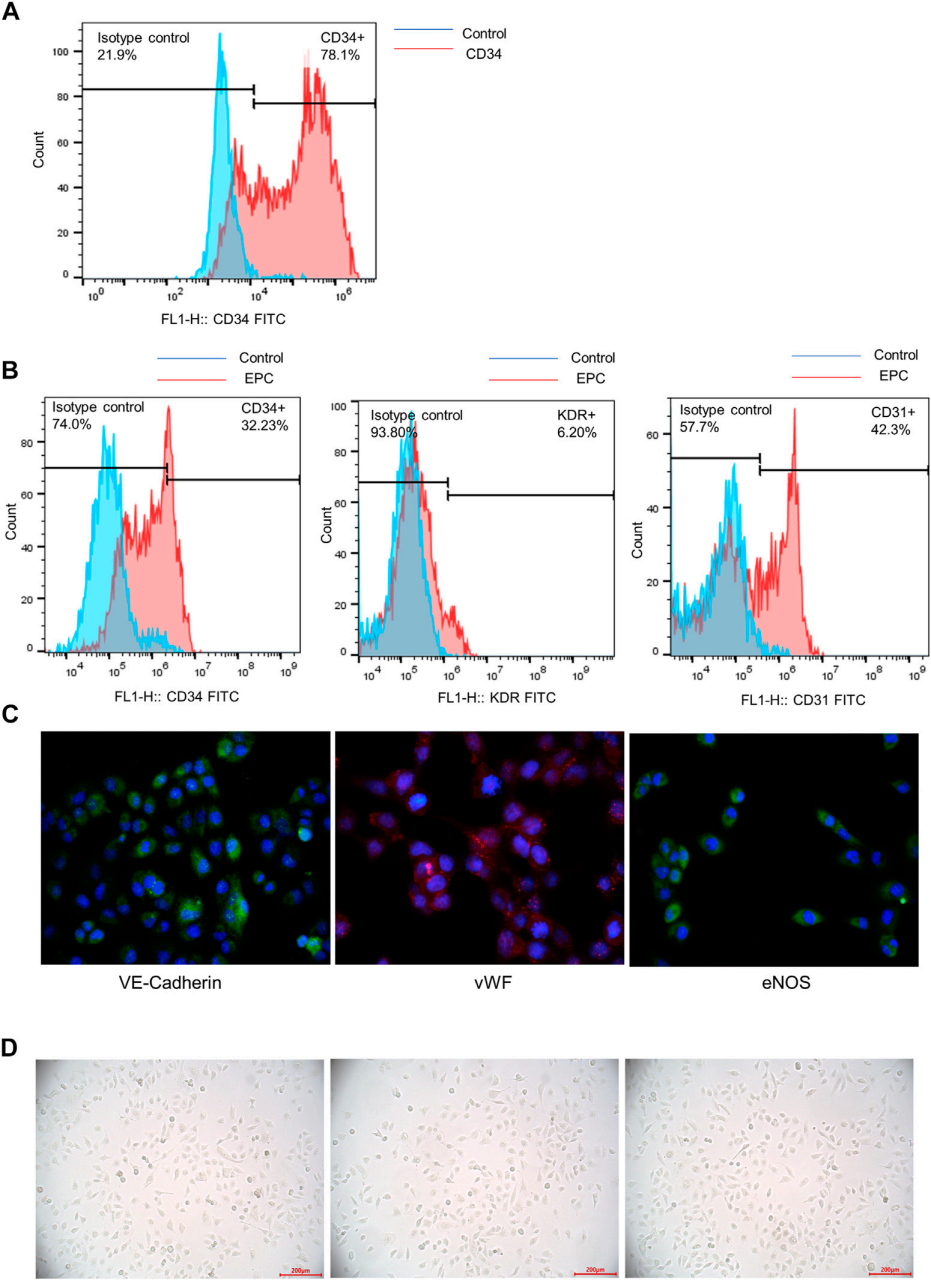
Identification of CD34^+^ hematopoietic stem cells and differentiation of CD34^+^ hematopoietic stem cells to early EPC. **(A)** The purity of CD34^+^ hematopoietic stem cells was accessed by a flow cytometry analysis. **(B)** Flow cytometry analysis was used to detect the expression of surface markers (CD34, KDR and CD31) of EPCs derived from CD34^+^ cells. **(C)** The immunofluorescence staining was used to examine the specific biomarkers of the endothelial cells on the cell surface (VE-Cadherin, vWF and eNOS). **(D)** The morphological features of EPCs derived from CD34^+^ hematopoietic stem cells were observed using a microscope.

Flow cytometry analysis was used to detect the purity of CD34^+^ hematopoietic stem cells. The result showed that nearly 80% of the cells expressed CD34 after positive selection process (Figure 1A). CD34^+^ hematopoietic stem cells were successfully expanded *in vitro*.

### Differentiation of CD34^+^ Hematopoietic Stem Cells to Early EPCs

Flow cytometry analysis showed the expression of surface markers of EPCs derived from CD34^+^ cells. The result indicated that CD34, KDR and CD31 were positively expressed in early EPCs. The expression of CD34 and KDR was relatively lower than CD31 expression (Figure 1B). The immunofluorescence staining of specific cell surface biomarkers of the endothelial cells including VE-Cadherin, vWF and eNOS were shown in Figure 1C. VE-Cadherin (green), vWF (red), eNOS (green) were expressed in early EPCs, and the cell nucleus was stained blue. The morphology of EPCs was observed under a microscope. The adherent cells were in spindle shape, which showed the morphological features of early EPCs cultured *in vitro* (Figure 1D). Overall, the CD34^+^ hematopoietic stem cells incubated *in vitro* were successfully differentiated into early EPCs.

### The Expression of Inflammatory Cytokines After LPC Treatment in EPCs

**FIGURE 2:**
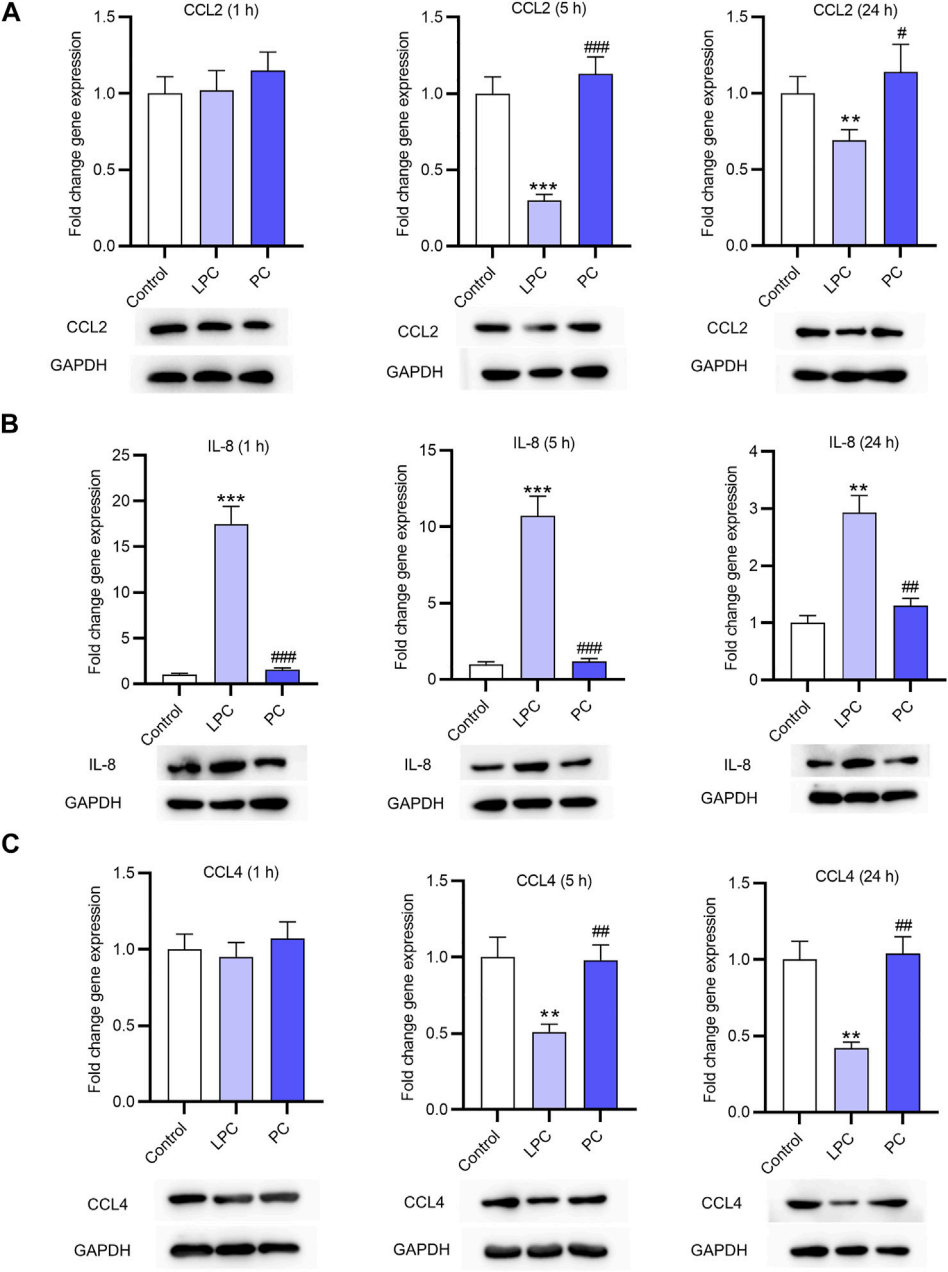
The expression of inflammatory cytokines after LPC or PC treatment in EPCs. RT-qPCR and western blotting analyses were used to detect the expression of inflammatory factors including CCL2 **(A)**, IL-8 **(B)**, CCL4 **(C)** in EPCs after 10 μg/ml of LPC or PC treatment for 1, 5 or 24 h **p < 0.01, ***p < 0.001 compared with the control, ^#^ *p* < 0.05, ^###^ *p* < 0.001 compared with the LPC group.

After treatment of LPC or PC at the concentration of 10 μg/ml for 1, 5 or 24 h, the EPCs were harvested and the expression levels of inflammatory factors (CCL2, IL-8, CCL4) in EPCs were detected using RT-qPCR analysis. The result showed that the expression of CCL2 was not significantly altered after treatment for 1 h compared with that in the control group. CCL2 mRNA expression was decreased by LPC by 70% after 5 h and by 31% after 24 h. CCL2 protein expression was also decreased by LPC after 5 and 24 h (Figure 2A). The IL-8 expression at the mRNA level was significantly higher in LPC group than in control group after 1 h (17.5 fold changes), 5 h (10.7 fold changes) and 24 h (2.9 fold changes). IL-8 protein expression was also increased after LPC treatment for 1, 5, 24 h (Figure 2B). Treatment of LPC for 1 h had no significant effects on CCL4 expression. CCL4 mRNA expression was significantly decreased by LPC by 49% for 5 h, by 58% for 24 h. CCL4 protein expression was also decreased by LPC after 5 and 24 h (Figure 2C).

### The Expression of Factors Associated With Angiogenesis After LPC Treatment in EPCs

**FIGURE 3:**
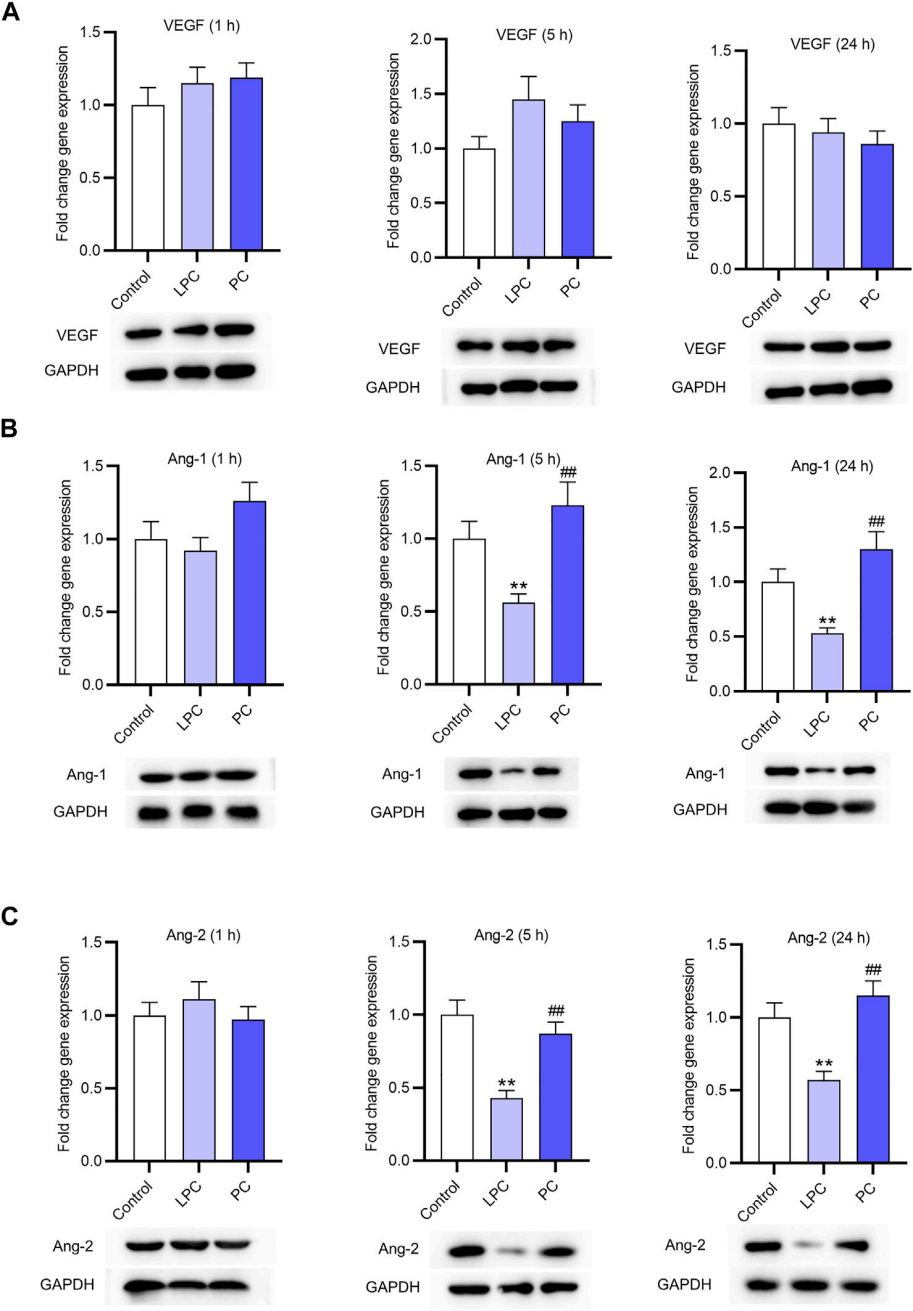
The expression of factors associated with angiogenesis after LPC or PC treatment in EPCs. RT-qPCR and western blotting was used to access the levels of angiogenesis-associated genes including VEGF **(A)**, Ang-1 **(B)**, Ang-2 **(C)** in EPCs after 10 μg/ml of LPC or PC treatment for 1, 5 and 24 h *p < 0.05, **p < 0.01 compared with the control, ^##^ *p* < 0.01 compared with the LPC group.

The expression of VEGF and Ang-1 in EPCs was accessed using RT-qPCR. After treatment for 1, 5 and 24 h, the expression of VEGF showed no significant changes (Figure 3A). Ang-1 and Ang-2 expression at the mRNA and protein levels was not significantly affected by LPC or PC treatment for 1 h. After treatment for 5 and 24 h, the Ang-1 and Ang-2 expression at the mRNA and protein levels was significantly lower in LPC group than in control group or PC group (Figures 3B,C). In detail, LPC treatment for 5 h decreased Ang-1 mRNA expression by 44%, and the number for 24 h is 47%. Ang-2 mRNA expression was inhibited by LPC treatment for 5 h by 57% and for 24 h by 43%.

### The Suppressive Effect of LPC on the Proangiogenesis of EPCs

**FIGURE 4:**
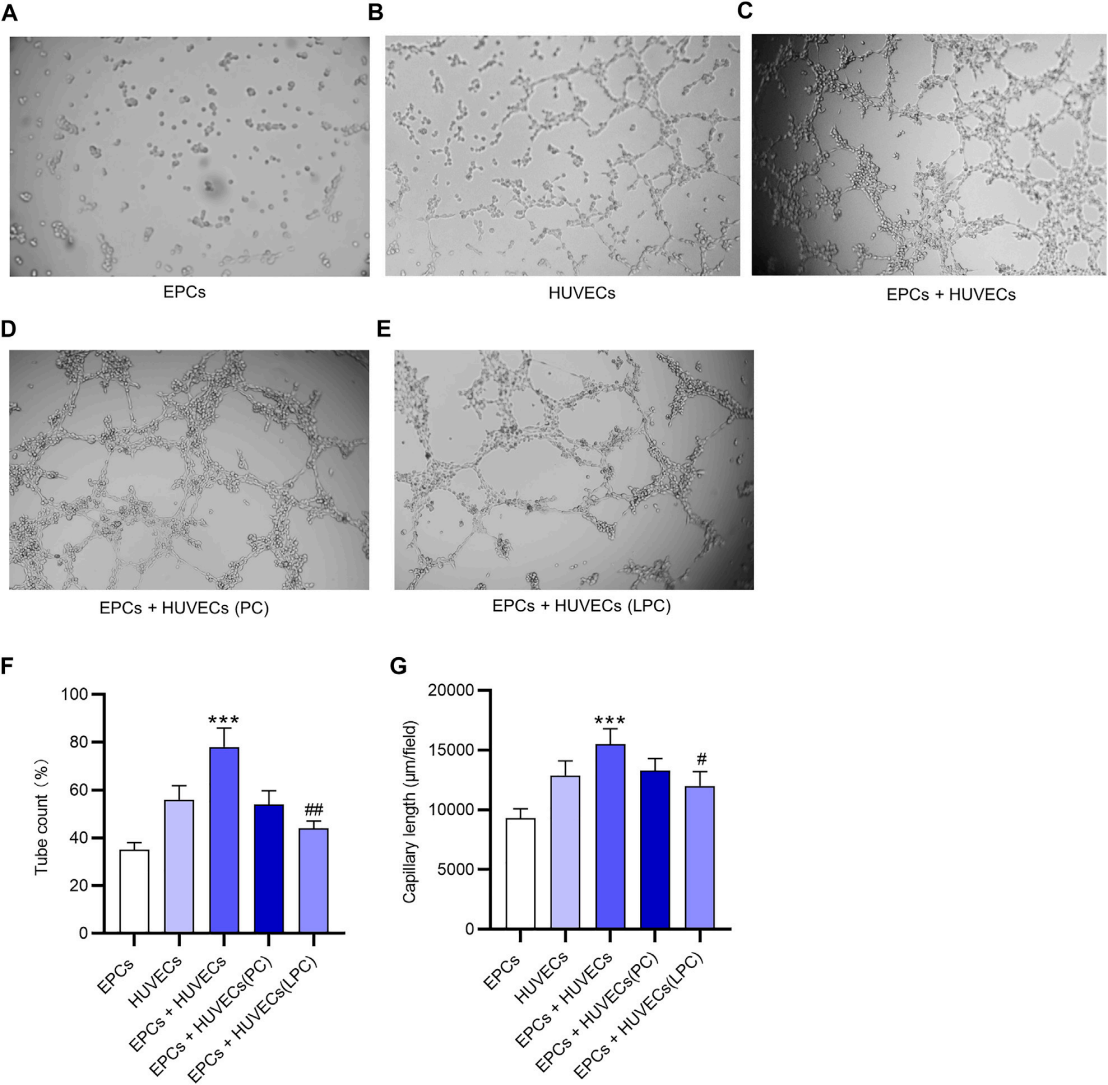
The suppressive effect of LPC on the proangiogenesis of EPCs. **(A)** A tube formation assay was used to examine the angiogenesis potential of EPCs *in vitro*. **(B)** Tubes developed by HUVECs *in vitro*. **(C)** Tubes developed by HUVECs cultured with the supernatant of EPCs *in vitro*. **(D)** After the PC treatment or **(E)** LPC treatment, the tubes formed by HUVECs that were cultured with supernatant of EPCs. **(F,G)** Tube count and capillary length of tubes in each group. ***p < 0.001 compared with the HUVECs group, ^#^ *p* < 0.05, ^##^ *p* < 0.01 compared with the EPCs + HUVECs group.

A tube formation assay was used to examine the angiogenesis potential of EPCs and HUVECs *in vitro*. The result demonstrated that EPCs alone did not independently develop tubes *in vitro* (Figure 4A). After incubation with supernatant of EPC cultures, HUVECs showed the increased potential of angiogenesis (Figures 4B,C). After LPC treatment, the tubes formed by HUVECs were loose and not intact compared with that after PC treatment (Figures 4D,E). These conclusions are supported by the data of quantification of tube count and capillary length, as revealed in Figures 4F,G.

### The Promotive Effect of LPC on the Activation of PKC Signaling in EPCs

**FIGURE 5:**
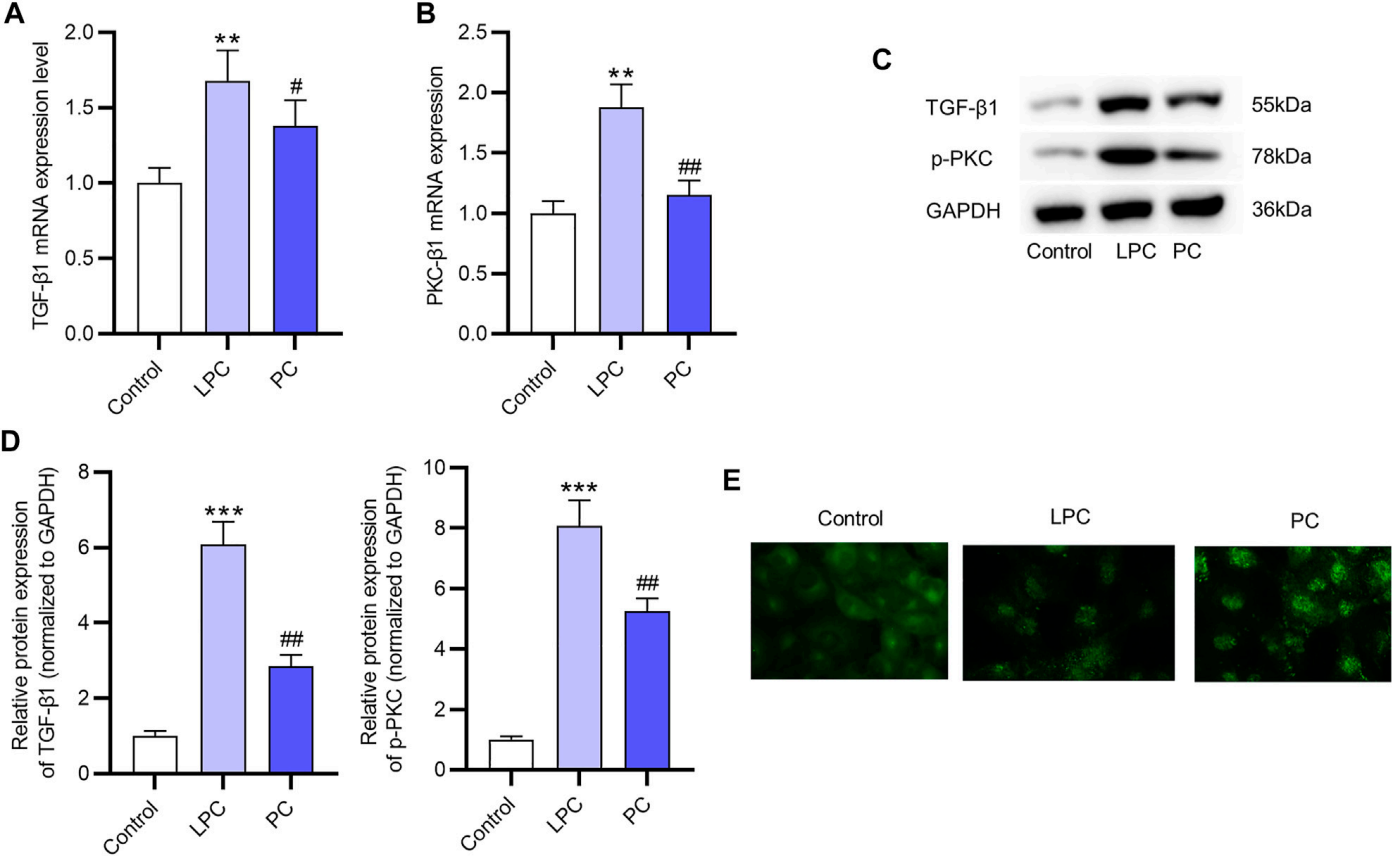
The promotive effect of LPC on the activation of the PKC signaling in EPCs. **(A,B)** The mRNA expression of PKC-β1 and TGF- β1 after LPC or PC treatment in EPCs were measured by RT-qPCR. **(C,D)** The protein levels of p-PKC and TGF-β1 were in EPCs treated with LPC or PC were accessed using western blot. **(E)** Immunofluorescence staining was used to determine the location of PKC-β1 after the LPC or PC treatment in EPCs (Figure 5D). **p < 0.01, ***p < 0.001 compared with the control, ^#^ *p* < 0.05, ^##^ *p* < 0.01 compared with the LPC group.

The mRNA levels of PKC-β1 and TGF- β1 in EPCs were both significantly increased after the LPC treatment compared with the control group (Figures 5A,B). The protein expression of p-PKC and TGF-β1 was higher in LPC group compared with the control group (Figures 5C,D). Moreover, according to results of immunofluorescence staining assay, PKC-β1 was primarily located in the cytoplasm, and the translocation of the PKC-β1 protein to the nucleus was promoted after LPC treatment in EPCs (Figure 5E).

## Discussion

EPCs, smaller than 15 μm, can be detected in blood and bone marrow. Since the discovery of EPCs in 1997 (Werner et al., 2003), the exploration of their functions and application in cell therapy has achieved great progress. *In vivo* assays showed that EPCs promoted angiogenesis and facilitated the repair of vascular injury (Werner et al., 2003). The differentiation potential of EPCs into cardiomyocytes and smooth muscle cells has also been reported, which suggests the beneficial effect of EPCs on myocardial function (Iwasaki et al., 2006; Jujo et al., 2008; Mudyanadzo, 2018). Lower level of circulating EPCs indicates a higher risk in patients with cardiovascular diseases (Tepper et al., 2005; Iwakura et al., 2006; Westerweel et al., 2008). Dysfunctions of EPCs are found in patients with some diseases such as diabetes (Dai et al., 2017), hypertension (Garcia et al., 2020) and obesity (Peterson et al., 2019).

In the present study, we cultured CD34^+^ peripheral blood hematopoietic stem cells and induced their differentiation into EPCs. EPCs are divided into two categories, early EPCs and late EPCs. The early EPCs are adherent spindle-shaped with limited proliferation ability. The EPCs in this study with similar phenotypes and functions to early EPCs. After differentiation, EPCs expressed specific surface membrane markers such as CD34, CD31, KDR. Immunofluorescence staining indicated that VE-Cadherin, vWF, eNOS were all positively expressed in spindle-shaped EPCs. Previous studies have revealed that CD31, KDR, VE-Cadherin, vWF and eNOS are specific surface markers of endothelial cells and indicate the differentiation of ECs to a more mature stage (Fish and Marsden, 2006; Lagarkova et al., 2008). Additionally, the function of EPCs in this study was further explored. We demonstrated that EPCs alone did not develop into integrated tubes, while addition of supernatants of EPCs promoted the angiogenesis of HUVECs.

LPC, a bioactive lipid molecule, is implicated in various biological processes such as cell proliferation (Rikitake et al., 2000; Schaefer et al., 2004), inflammation (Gonçalves et al., 2012) and angiogenesis (Murugesan and Fox, 1996). Additionally, previous studies have revealed that LPC activates protein kinase C (PKC) in endothelial cells and platelets (Kugiyama et al., 1992). In the present study, LPC was demonstrated to inhibit the expression of angiogenesis-related factors including Ang-1 and Ang-2 at the mRNA and protein levels *in vitro* but had no significant effects on VEGF expression. We found that LPC treatment for 5 and 24 h suppressed the mRNA and protein expression of CCL2, CCL4, but induced the expression of IL-8. Many reports revealed that IL-8 serves as an angiogenesis promoter and induces tube formation (Bancroft et al., 2001; Dimberg, 2010; Lattanzio et al., 2013). Contradictorily, Yifat Amir Levy et al. reported that IL-8 decreases tube number or capillary outgrowth of HUVECs (Amir Levy et al., 2015). The angiogenesis of HUVECs promoted by supernatant of EPCs was demonstrated to be inhibited by the LPC treatment. Moreover, our findings revealed that LPC activated the PKC signaling pathway, which is supported by other studies (Awasthi et al., 2016; Tseng et al., 2019). IL-8 can activate the PKC signaling pathway (Xiao et al., 2018), which is closely associated with the impaired tube-forming ability of the HUVECs (Huang et al., 2015). The upregulated IL-8 expression and the diminished tubes formed by HUVECs under LPC treatment in the present study might be associated with the activation of the PKC pathway.

In conclusion, our study innovatively revealed that LPC suppressed expression of pro-inflammatory cytokines including CCL2 and CCL4, inhibited expression of proangiogenic factors including Ang-1 and Ang-2 in early EPCs differentiated from CD34^+^ peripheral blood hematopoietic stem cells in a PKC pathway dependent manner. Supernatant of EPCs facilitates the tube formation ability of HUVECs. The present study may provide clues for the application of EPC therapy in angiogenesis-related diseases, especially some cardiovascular diseases. To optimize its therapeutic outcomes, our understanding of the mechanisms by which EPCs contribute to tissue repair must be expanded, the methods of EPC purification, expansion, and administration need to be refined, and the techniques that overcome the scarcity and dysfunction of EPCs need to be developed. Several limitations of the current work merit mention. First, we used a traditional method for the analysis of relative quantification of PCR data in the present study, while some novel methods (Yuan et al., 2006; Steibel et al., 2009) are more applicable for the linear models. Second, *in vivo* studies are needed to further validate the therapeutic value of EPCs on proangiogenesis.

## Data Availability

The original contributions presented in the study are included in the article/Supplementary Material, further inquiries can be directed to the corresponding author.
